# Prevalence and risk factors of gestational diabetes mellitus: findings from a universal screening feasibility program in Lima, Peru

**DOI:** 10.1186/s12884-018-1904-0

**Published:** 2018-07-18

**Authors:** Gloria T. Larrabure-Torrealva, Stephanie Martinez, Miguel Angel Luque-Fernandez, Sixto E. Sanchez, Pedro A. Mascaro, Hugo Ingar, Walter Castillo, Rina Zumaeta, Mirtha Grande, Vicky Motta, Percy Pacora, Bizu Gelaye, Michelle A. Williams

**Affiliations:** 1 Instituto Nacional Materno Perinatal de Lima, Lima, Perú; 2 Departamentos de Medicina y Ginecología y Obstetricia, Universidad Nacional Mayor de San Marcos, Lima, Perú; 3 Department of Epidemiology, Harvard T.H. Chan School of Public Health, 677 Huntington Avenue, Kresge 505F, Boston, MA 02115 USA; 4 Asociación Civil Proyectos en Salud, A.C. PROESA, Lima, Perú; 5 Facultad de Medicina, Universidad Particular San Martin de Porres, Lima, Peru

**Keywords:** Diabetes mellitus, Gestational diabetes mellitus, Obesity, Depression, Pregnancy, IADPSG criteria, Peru

## Abstract

**Background:**

Gestational diabetes mellitus (GDM) is a global public health concern with potential implications for the health of a mother and her offspring. However, data on the prevalence and risk factors of GDM in Latin America are scarce.

The study was designed to estimate the prevalence of GDM and identify maternal risk factors among Peruvian women.

**Methods:**

A cross-sectional study was conducted among 1300 pregnant women attending a prenatal clinic in Lima, Peru. GDM was diagnosed using an Oral Glucose Tolerance Test (OGTT) performed between 24 and 28 gestational weeks using the International Association of Diabetes and Pregnancy Study Groups (IADPSG) criteria. Depression status was assessed using the Patient Health Questionnaire-9. Multivariate logistic regression models were used to identify risk factors of GDM.

**Results:**

Approximately 16% of pregnant women were diagnosed with GDM. The prevalence of obesity and depression were 24.4 and 10.6%, respectively. After adjusting for confounders, mid-pregnancy obesity was associated with a 1.64-fold increased odds of GDM (OR: 1.64; 95% CI: 1.03–2.61). Participants with a family history of diabetes had a 1.5-fold increased odds of developing GDM (OR: 1.51, 95% CI: 1.10–2.07) as compared to women without this family history. Depression was associated with a 1.54-fold increased odds of GDM (OR: 1.54; 95% CI:1.09–2.17).

**Conclusions:**

GDM is highly prevalent and was associated with maternal obesity, family history of diabetes and antepartum depression among Peruvian women. Intervention programs aimed at early diagnoses and management of GDM need to take maternal obesity, family history of diabetes and antepartum depression into account.

**Electronic supplementary material:**

The online version of this article (10.1186/s12884-018-1904-0) contains supplementary material, which is available to authorized users.

## Background

Gestational diabetes mellitus (GDM) is a non-communicable disease affecting pregnant women [1, 2]. Globally the median estimates of GDM range from 6 to 13% [2]. In the United States, recent estimates show up to 9% of all pregnancies are complicated by GDM [3]. In Central and South America, the recent overall prevalence of GDM is estimated at 11% [2]. There is well-established evidence showing that women with GDM are at risk for preeclampsia [4, 5], premature birth [6], increased risk of cesarean section [4, 5] and later development of type 2 diabetes [2]. GDM is also associated with increased risk of perinatal complications including malformations [7], shoulder dystocia [5], neonatal hypoglycemia [8], and perinatal mortality [8, 9].

Obesity and a family history of diabetes have been consistently identified as major risk factors for GDM in previous studies [3]. Other risk factors for GDM include advanced maternal age [10], nonwhite race [10, 11], previous unexplained stillbirth [12, 13], and obesity [10, 13]. In addition to increased GDM risk, maternal obesity increases the risk of multiple adverse maternal health outcomes including thrombosis [14], gestational hypertension [15], preeclampsia [16–18], preterm delivery [19], and cesarean section [16, 18]. Of note, significant neonatal complications have been associated with obesity and GDM. These include congenital anomalies [20, 21], macrosomia [22], and birth injury [23, 24]. An expanding body of evidence now implicates unipolar major depressive disorder as one of the major risk factors for and conditions co-occurring with GDM [25], although the evidence is inconsistent [26].

Peru, a middle-income country, is one of the countries experiencing an epidemiologic transition with increasing burden of non-communicable disease risk factors [27, 28]. A 2014 national survey in Peru of people ≥15 years old showed the prevalence of overweight is 35% and the prevalence of obesity is 18% of the population [29]. However, the prevalence of GDM and its risk factors have not been investigated in Peru. Given this gap in the literature and the increased burden of non-communicable diseases in the country, we sought to examine the prevalence of GDM and associated risk factors in a cohort of pregnant women in Lima, Peru.

## Methods

### Study population

The Screening Treatment and Effective Management of Gestational Diabetes Mellitus (STEM-GDM) was designed to evaluate the prevalence of GDM among Peruvian women attending perinatal care and to provide evidence to improve the local guidelines for standardized GDM screening/diagnosis, effective management, and treatment. Participants were 1300 pregnant women attending prenatal care clinic at the Instituto Nacional Materno Perinatal (INMP) in Lima, Peru (Additional file 1). Recruitment occurred between February 2013 and June 2014. The Instituto Nacional Materno Perinatal (INMP), overseen by the Peruvian Ministry of Health, is the primary referral center for maternal and perinatal care in Lima, Peru.

Women were eligible if they were at least 18 years of age, had a gestational age of 24 to 28 gestational weeks, and could speak, read and write Spanish. Participants were excluded if they planned to deliver at another hospital or location, date of last menstrual period not certain and not confirmed by ultrasound exam performed prior to 24 weeks of gestation, unable to complete an oral glucose tolerance test (OGTT), known to have a multiple pregnancy, had a previous diagnosis of overt diabetes requiring treatment with medication before the pregnancy, or were currently receiving medical treatment for chronic diseases, such as oral glucocorticoids, thiazide diuretics, β-blockers, ACE inhibitors, oral β-mimetics, Dilantin, or antiretroviral agents. All participants provided written informed consent. Study procedures were approved by institutional review boards of the INMP, Lima, Peru and the Harvard T.H. Chan School of Public Health Office of Human Research Administration, Boston, MA, USA.

### Gestational Diabetes Mellitus (GDM) assessment

GDM cases were identified using the OGTT, which is considered the gold standard for this diagnosis. For the OGTT test, participants were appointed to the laboratory at INMP between 8 AM to 9 AM after 8-h overnight fasting. A trained lab technician obtained a basal venous blood sample of 15cm^3^ for measuring fasting plasma glucose (PG). Participants were administered 75 g of anhydrous oral glucose dissolved in 250 ml of water. After 1 and 2 h, new venous blood samples were obtained for measuring PG levels. Plasma glucose value was measured by the glucose oxidase-peroxidase method in duplicate using an auto-analyzer for biochemical tests. Standard clinical laboratory quality control assessment of the OGTT testing was completed to assure reliability and validity of measured plasma glucose.

### Interpretation of the OGTT

According to the standard OGTT testing procedures outlined by the IADPSG recommendations [30], pregnant women were considered to have GDM if fasting plasma glucose (PG) was ≥92 mg/dl (5.1 mmol/l), 1 h-h PG ≥180 mg/dl (10 mmol/l), and/or 2-h PG ≥153 mg/dL (≥8.5 mmol/L). Overall, 205 women were diagnosed with GDM. Of the GDM diagnosed women, 186 women (90.7%) had a fasting PG ≥ 92 mg/dl, 29 women (14.1%) had a 1-h PG ≥180 mg/dl, and 24 women (11.7%) had a 2-h PG ≥153 mg/dL. Women diagnosed with GDM received glucometers, nutritional advice, and clinical care to manage their condition.

### Sociodemographic characteristics

Women were interviewed using a structured questionnaire to collect detailed information on sociodemographic, lifestyle characteristics, medical and reproductive history. Sociodemographic information collected included participants’ age, family history of diabetes mellitus among first degree-relatives, body weight, height, smoking status, and alcohol use. Other covariates included maternal and paternal education (≤6, 7–12, > 12 years); ethnicity (Mestizo vs. others); marital status (married/living with partner vs. others); employment status (employed vs. not employed); difficulty paying for the basics (very hard/hard, somewhat hard, not very hard); difficulty accessing medical care (very hard/hard, somewhat hard, not very hard); food insecurity (no vs. yes), and perceived health during pregnancy. Pre-pregnancy body mass index (BMI) and mid-pregnancy BMI were calculated as weight (in kilograms) divided by the square of height (in meters and used to identify normal weight (BMI < 25 kg/m^2^), overweight (BMI: 25–29.9 kg/m^2^), and obese (BMI ≥ 30 kg/m^2^)) women. Due to the small number of underweight women, this group was merged with normal weight women. For the purpose of our analysis, obesity was grouped into a single category of BMI of 30.0 kg/m^2^ and higher. Depression status was assessed using the 9-item Spanish-language Patient Health Questionnaire-9 (PHQ-9) [31, 32], which has been validated in a population of pregnant Peruvian women [33]. The PHQ-9 score was calculated by assigning a score between 0 and 3 to the response categories of “not at all,” “several days,” “more than half the days,” and “nearly every day.” Depression was defined as a PHQ-9 score ≥ 10.

### Statistical analysis

Sociodemographic characteristics were evaluated using numbers (%) for categorical variables and means (± standard deviations) for continuous variables. Chi-square tests and Student’s t-tests were conducted to compare distributions between women with and without GDM. Prevalence estimates were determined for GDM in relation to socio-demographic and behavioral characteristics. Adjusting for covariates of interest, we used multivariable logistic regression procedures to calculate odds ratios (OR) and 95% confidence intervals (95% CI) to estimate associations of GDM with risk factors. Forward logistic regression modeling procedures combined with the change-in-estimate approach were used to select the final report models [34]. Variables of a priori interest (e.g., age and BMI) were forced into final models. All analyses were performed using IBM’s SPSS Statistical Software for Windows (IBM SPSS Version 22, Chicago, Illinois, USA). All reported *p*-values are two-sided and deemed statistically significant at α = 0.05.

## Results

**Fig. 1:**
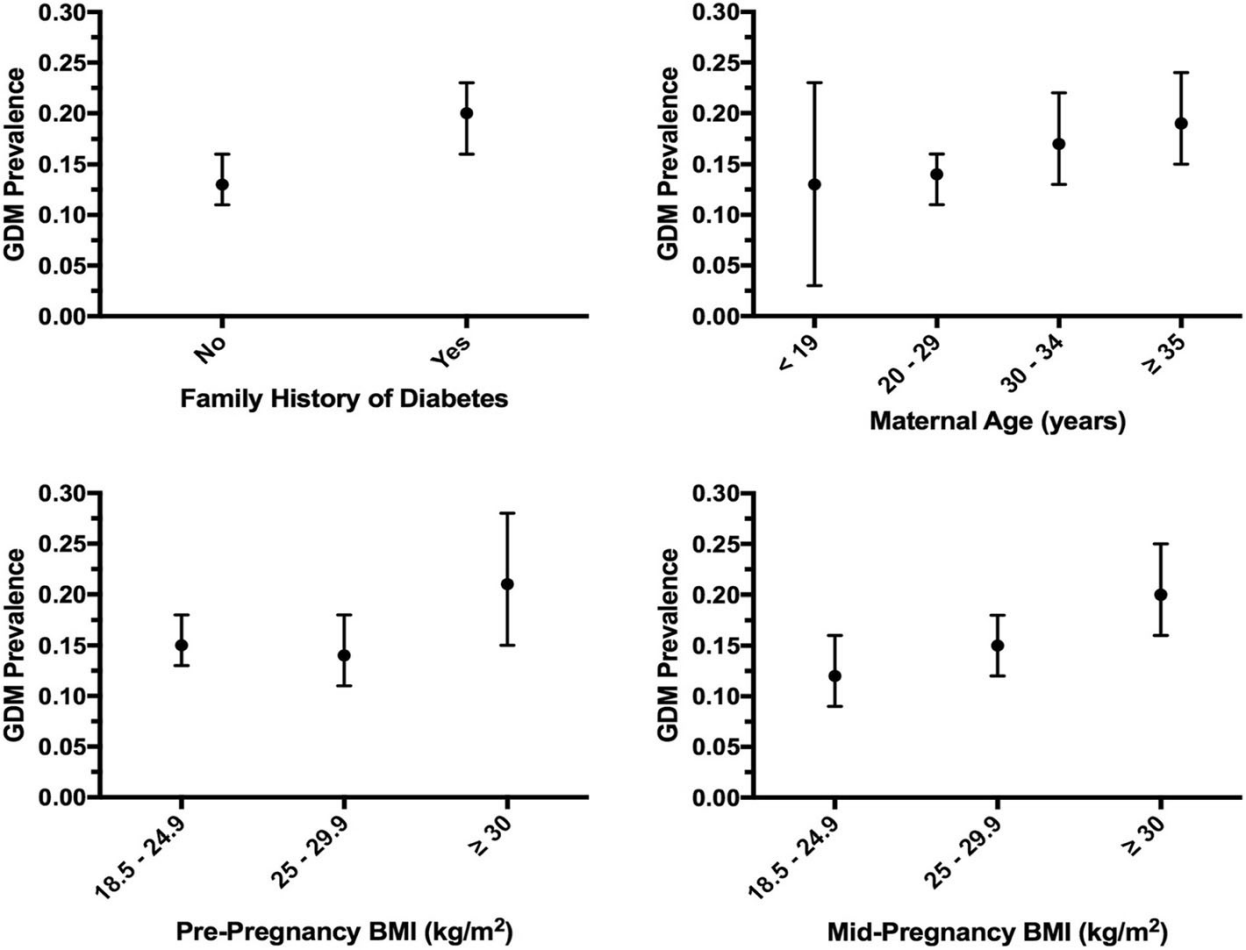
Prevalence of GDM by family history of diabetes mellitus among first degree-relatives, maternal age, pre-pregnancy BMI, and mid-pregnancy BMI. Point estimates and 95%CI are reported

Maternal sociodemographic and reproductive characteristics of the study population are presented in Table 1. The average age of study participants was 28.9 years (± 6.1). A majority of participants were married or living with a partner (86.5%), Mestizo (98.1%), and unemployed (67.8%) (Table 1). The overall prevalence of GDM in our population of Peruvian pregnant women was 15.8%. The prevalence of mid-pregnancy obesity was 24.5% while 10.6% of them had depression. Compared to women without GDM, women with GDM were significantly older (*p* = 0.013), more likely to report a family history of diabetes (*p* = 0.005), have higher mid-pregnancy BMI (*p* = 0.016) and have depression (*p* = 0.012). The prevalence of GDM increased significantly with the presence of family history of diabetes mellitus (p = 0.005) and maternal mid-pregnancy obesity (*p* = 0.02) (Fig. 1).

**Table 1:** Maternal sociodemographic characteristics (*N* = 1300)

Characteristics	All participants (*N* = 1300)		No GDM (*N* = 1095)		GDM (*N* = 205)		*P-*value
	n	%	n	%	n	%	
Maternal age (years)^a^	28.86 ± 6.14		28.68 ± 6.06		29.83 ± 6.49		*0.013*
Maternal age (years)							
< 19	49	3.8	43	3.9	6	2.9	0.081
20–29	669	51.5	578	52.8	91	44.4	
30–34	313	24.1	258	23.6	55	26.8	
≥35	269	20.7	216	19.7	53	25.9	
Ethnicity							
Mestizo	1275	98.1	1074	98.1	201	98.0	0.974
Other	25	1.9	21	1.9	4	2.0	
Marital status							
Married or living with partner	1123	86.5	946	86.5	177	86.3	0.960
Single or living alone/divorced	176	13.5	148	13.5	28	13.7	
Educational Status (years)	12.17 ± 2.38		12.16 ± 2.39		12.25 ± 2.33		0.604
Maternal education (years)							
≤6	39	3.0	34	3.1	5	2.4	0.743
7–12	698	53.7	591	54.0	107	52.2	
> 12	563	43.3	470	42.9	93	45.4	
Paternal education (years)							
≤6	30	2.3	27	2.5	3	1.5	0.318
7–12	727	56.4	620	57.0	107	53.0	
> 12	532	41.3	440	40.5	92	45.5	
Employment							
No	881	67.8	733	66.9	148	72.2	0.140
Yes	419	32.2	362	33.1	57	27.8	
Pre-pregnancy BMI (kg/m^2^)							
< 25 (normal weight)	684	53.6	580	53.8	104	52.3	0.137
25–29.9 (overweight)	446	34.9	382	35.4	64	32.2	
≥30 (obese)	147	11.5	116	10.8	31	15.6	
Mid-pregnancy BMI (kg/m^2^)							
< 25 (normal weight)	324	25.0	284	26.1	40	19.5	*0.016*
25–29.9 (overweight)	653	50.5	553	50.8	100	48.8	
≥30 (obese)	317	24.5	252	23.1	65	31.7	
Family history of diabetes							
No/Don’t Know	834	64.2	720	65.8	114	114	*0.005*
Yes	466	35.8	375	34.2	91	44.4	
Depression	138	10.6	106	9.7	32	15.6	*0.012*
Perceived health during pregnancy							
Excellent	18	1.4	14	1.3	4	2.0	0.696
Very Good	76	5.8	67	6.1	9	4.4	
Good	586	45.1	488	44.6	98	47.8	
Fair	568	43.7	483	44.1	85	41.5	
Poor	52	4.0	43	3.9	9	4.4	
Difficulty paying for basics							
Very hard/hard	199	15.3	171	15.6	28	13.7	0.759
Somewhat hard	441	34.0	369	33.8	72	35.1	
Not very hard	658	50.7	553	50.6	105	51.2	
Food insecurity							
No	446	34.3	379	34.6	67	32.7	0.593
Yes	854	65.7	716	65.4	138	67.3	
Difficulties to access medical care							
Very hard/hard	248	19.1	207	18.9	41	20.0	0.728
Somewhat hard	897	69.1	760	69.5	137	66.8	
Not very hard	154	11.9	127	11.6	27	13.2	

^a^mean ± standard deviation

Italicized *p*-values are statistically significant at α = 0.05

In an unadjusted bivariate analysis, participants with a family history of diabetes had 1.53-fold increased risk of GDM (OR: 1.53, 95%CI: 1.13–2.07), and participants with mid-pregnancy obesity had 1.83-fold increased risk of GDM compared to those of normal weight (OR: 1.83, 95%CI: 1.19–2.81) (Table 2). Compared to non-depressed participants, those with depression had a 1.5-fold increased odds of GDM (OR: 1.52, 95%CI: 1.09–2.12). After adjusting for age and family history of diabetes, the association between depression status and GDM remained significant (OR: 1.53, 95%CI: 1.09–2.14). In a multivariate model, a family history of diabetes remained significantly associated with GDM (OR: 1.51, 95%CI: 1.10–2.07), mid-pregnancy obesity slightly increased the risk for GDM (OR: 1.64, 95%CI: 1.03–2.61), and depression was also significantly associated with GDM (OR: 1.54, 95%CI: 1.09–2.17) (Table 2).

**Table 2:** Prevalence and risk factors of gestational diabetes (*N* = 1300)

Characteristics	Crude OR (95%CI)	Age and family history of diabetes adjusted OR (95%CI)^a^	Multivariate OR (95%CI)^b^
Maternal age (years)	1.03 (1.01–1.06)	–	1.03 (1.00–1.06)
Ethnicity			
Mestizo	Reference	Reference	Reference
Other	1.02 (0.35–3.00)	1.05 (0.35–3.10)	1.01 (0.34–3.02)
Marital status			
Married or living with partner	Reference	Reference	Reference
Single or living alone/divorced	1.01 (0.66–1.56)	1.10 (0.70–1.71)	1.04 (0.66–1.66)
Maternal education (years)			
≤6	Reference	Reference	Reference
7–12	1.23 (0.47–3.22)	0.79 (0.3–2.11)	1.13 (0.42–3.03)
> 12	1.35 (0.51–3.53)	0.92 (0.69–1.27)	1.13 (0.41–3.10)
Partner education (years)			
≤6	Reference	Reference	Reference
7–12	1.55 (0.46–5.21)	0.52 (0.15–1.76)	1.72 (0.50–5.88)
> 12	1.88 (0.56–6.34)	0.85 (0.63–1.16)	1.98 (0.57–6.86)
Pre-pregnancy BMI (kg/m^2^)			
< 25 (normal weight)	Reference	Reference	Reference
25–29.9 (overweight)	0.93 (0.67–1.31)	0.86 (0.61–1.21)	0.90 (0.63–1.27)
≥30 (obese)	1.49 (0.95–2.33)	1.22 (0.76–1.95)	1.29 (0.80–2.07)
Mid-pregnancy BMI (kg/m^2^)			
< 25 (normal weight)	Reference	Reference	Reference
25–29.9 (overweight)	1.28 (0.87–1.90)	1.18 (0.79–1.77)	1.23 (0.82–1.87)
≥30 (obese)	1.83 (1.19–2.81)	1.54 (0.98–2.41)	1.64 (1.03–2.61)
P-value for trend			0.033
Family history of diabetes			
No/Don’t know	Reference	–	Reference
Yes	1.53 (1.13–2.07)	–	1.51 (1.10–2.07)
Depression			
No	Reference	Reference	Reference
Yes	1.52 (1.09–2.12)	1.53 (1.09–2.14)	1.54 (1.09–2.17)
Perceived health during pregnancy			
Excellent	Reference	Reference	Reference
Very good	0.47 (.13–1.74)	0.52 (0.14–1.93)	0.62 (0.16–2.34)
Good	0.70 (0.23–2.18)	0.75 (0.24–2.35)	0.89 (0.28–2.82)
Fair	0.62 (0.20–1.92)	0.63 (0.20–1.97)	0.69 (0.22–2.21)
Poor	0.73 (0.20–2.75)	0.73 (0.19–2.76)	0.76 (0.20–2.92)
Difficulty paying for basics			
Very hard/hard	Reference	Reference	Reference
Somewhat hard	1.19 (0.74–1.91)	1.24 (0.77–1.99)	1.18 (0.73–1.93)
Not very hard	1.16 (0.74–1.82)	1.25 (0.79–1.97)	1.19 (0.74–1.91)

^a^Adjusted for age (continuous) and family history of diabetes mellitus among first degree-relatives (yes vs. no/don’t know)

^b^Main multivariate regression model adjusted for all characteristics (maternal age, ethnicity, marital status, maternal education, paternal education, mid-pregnancy BMI, family history of diabetes, depression, perceived health during pregnancy, and difficulty paying for the basics) except pre-pregnancy BMI and OR (95%CI) from this model are reported here. A second multivariate regression model adjusted for all covariates except mid-pregnancy BMI and the OR (95%CI) for pre-pregnancy BMI is reported from this model

## Discussion

To the best of our knowledge, this is the first study to examine the prevalence of GDM in Lima, Peru using the IADPSG criteria, as well as the presence of family history of diabetes mellitus, maternal mid-pregnancy obesity, and maternal depression as independent risk factors of GDM. Approximately 16% of pregnant women were diagnosed with GDM. The prevalence of obesity was 24% and the prevalence of depression was 11%. A previous study by Sacks et al. using IADPSG criteria and a multinational cohort (*n* = 23,957) reported the prevalence of GDM varies from 9 to 26% and is associated with older maternal age, BMI, family history of diabetes, mean OGTT glucose values, and ethnicity [35]. Globally, the prevalence of GDM varies by study setting and diagnostic criteria. Additional influencing factors include age, obesity, and use of health services. For instance, using International Classification of Diseases (ICD) diagnostic codes (ICD-9 and ICD-10), GDM prevalence was reported as 6% in the USA [36], and 10% in Korea [37]. A meta-analysis of 22 studies in sub-Saharan Africa with a range of diagnostic criteria and study setting reported a GDM prevalence of 2–6% [13]. A study in China using a 75-g 2-h OGTT reported a GDM prevalence of 8%. A higher percentage of GDM diagnoses were made by fasting PG than 1- or 2-h glucose levels compared to previous estimates.

We found a significant association between GDM and mid-pregnancy obesity (BMI > 25). Maternal age of 30 years or older and family history of diabetes were also significantly associated with GDM. Our results are in general agreement with previous studies that have identified similar associations with GDM risk factors. For example, Kumari et al.*,* found a GDM prevalence of 24.5% in obese compared to 2.2% in non-obese women (*P* < 0.0001) [38]. In a retrospective cohort study of 613 obese (BMI > 35; class II and III) and 11,313 non-obese women, there was a threefold increase of GDM for obese patients compared to non-obese women (OR: 3.2, 95%CI: 2.5–4.2) [39]. In a recent meta-analysis of published literature, Chu and colleagues reported ORs of developing GDM of 2.14 (95% CI: 1.82–2.53), 3.56 (95% CI: 3.05–4.21), and 8.56 (95% CI: 5.07–16.04) among overweight, obese, and severely obese compared with normal-weight pregnant women, respectively [40]. According to Kim et al. in a study of pregnant women from Florida, USA, the likelihood of GDM increased with increasing BMI was significant for all racial/ethnic groups investigated [41]. Additionally, the proportion of GDM cases attributable to overweight and obesity was 41.1% overall [41]. The HAPO Study Cooperative Research Group has demonstrated that higher maternal BMI is associated with increased likelihood of pregnancy complications, including complications related to fetal growth, adiposity, and preeclampsia [42].

There is a growing body of epidemiologic evidence that shows depression as a risk factor for GDM [43, 44]. Our study results showing a significant association between depression and GDM is in general agreement with some prior studies [25, 43] but not all [26]. Recently, a longitudinal study in the USA from Hinkle et al. found that persistently high depression scores in the 1st and 2nd trimesters were associated with three-fold increased risk of GDM (highest vs lowest quartile in both trimesters: RR: 3.21, 95% CI: 1.00–10.28) [43]. In a cross-sectional study, women with GDM were more than three times more likely to have a history of depression compared to non-depressed women (OR: 3.79, 95% CI: 1.07–13.45) [25]. However, using data from prenatal care clinics at a University of Washington Medical Center, Katon et al. found no evidence of association between GDM and antepartum depression (OR = 0.95; 95%CI: 0.68–1.33) [26]. In a addition, a 2016 meta-analysis shows there is no clear consensus on the association between depression and diabetes during pregnancy and additional studies are needed [45]. Although we do not have a clear explanation for these findings, we speculate that differences in nutrition, physical activity, and other lifestyle characteristics may have contributed to the observed differences. Pregnancy is a major life event that increases vulnerability to depression, and pregnant women who have comorbid depression and GDM with serious implications for both maternal and infant outcomes.

This study had several strengths, including the use of trained interviewers and standard laboratory procedures for measuring plasma glucose. Our relatively large sample size and the high prevalence of gestational diabetes give us statistical power to study the associations of interest. However, some limitations must be considered when interpreting the results of our study. First, a family history of diabetes was assessed based on self-report; therefore, we cannot rule out recall bias. Additionally, previous studies have shown associations between GDM and adverse pregnancy outcomes. However, information on pregnancy outcomes was not collected in our study cohort. Lastly, participants in this study were pregnant women living in Lima, and the results may not be generalizable to the whole Peruvian population. Future studies that collect objective measures potentially associated with GDM and obesity may overcome concerns about these potential errors.

## Conclusion

Overweight and obesity may be prevented by implementing a healthy lifestyle that includes physical activity and nutritional counseling before pregnancy. If women are already obese when they become pregnant, O’Dwyer et al. suggests early screening for women with a body mass index > 34.9 kg/m in order to optimize maternal glycemic control during pregnancy [46]. In a retrospective cohort study, Lutsic et al. found that the proportion of women with maternal or delivery complications was highest among overweight or obese depressed pregnant women [47]. These studies provide evidence of a high prevalence of GDM in women with an elevated mid-pregnancy BMI and depression. GDM is prevalent in this population, especially in overweight/obese and depressed pregnant women. Our findings may be used to develop and implement programs aimed at supporting high-risk mothers in antenatal care.

## Additional file

Additional file 1: Study flowchart. (DOCX 44 kb)

## Abbreviations

BMI: Body mass index
GDM: Gestational diabetes mellitus
IADPSG: International Association of Diabetes and Pregnancy Study Groups
INMP: Instituto Nacional Materno Perinatal
OGTT: Oral Glucose Tolerance Test
PHQ-9: Patient Health Questionnaire-9
STEM-GDM: Screening Treatment and Effective Management of Gestational Diabetes Mellitus

## References

[1] Ferrara A (2007). Increasing prevalence of gestational diabetes mellitus: a public health perspective. Diabetes Care.

[2] Zhu Y, Zhang C (2016). Prevalence of gestational diabetes and risk of progression to type 2 diabetes: a global perspective. Curr Diab Rep.

[3] DeSisto CL, Kim SY, Sharma AJ (2014). Prevalence estimates of gestational diabetes mellitus in the United States, Pregnancy Risk Assessment Monitoring System (PRAMS), 2007-2010. Prev Chronic Dis.

[4] Waters TP, Dyer AR, Scholtens DM, Dooley SL, Herer E, Lowe LP, Oats JJ, Persson B, Sacks DA, Metzger BE (2016). Maternal and neonatal morbidity for women who would be added to the diagnosis of GDM using IADPSG criteria: a secondary analysis of the hyperglycemia and adverse pregnancy outcome study. Diabetes Care.

[5] Fadl HE, Ostlund IK, Magnuson AF, Hanson US (2010). Maternal and neonatal outcomes and time trends of gestational diabetes mellitus in Sweden from 1991 to 2003. Diabet Med.

[6] Hedderson M (2003). Gestational diabetes mellitus and lesser degrees of pregnancy hyperglycemia: association with increased risk of spontaneous preterm birth. Obstet Gynecol.

[7] Sheffield J, Butler-Koster E, Casey B, McIntire D, Leveno K (2002). Maternal diabetes mellitus and infant malformations. Obstet Gynecol.

[8] Bartha JL, Martinez-Del-Fresno P, Comino-Delgado R (2000). Gestational diabetes mellitus diagnosed during early pregnancy. Am J Obstet Gynecol.

[9] Wood S, Jick H, Sauve R (2003). The risk of stillbirth in pregnancies before and after the onset of diabetes. Diabet Med.

[10] Solomon C, Willett W, Carey V, Rich-Edwards J, Hunter D, Colditz G, Stampfer M, Speizer F, Spiegelman D, Manson J (1997). A prospective study of pregravid determinants of gestational diabetes mellitus. JAMA.

[11] Hunsberger M, Rosenberg KD, Donatelle RJ (2010). Racial/ethnic disparities in gestational diabetes mellitus: findings from a population-based survey. Womens Health Issues.

[12] McMahon M, Ananth C, Liston R (1998). Gestational diabetes mellitus. Risk factors, obstetric complications and infant outcomes. J Reprod Med.

[13] Mwanri AW, Kinabo J, Ramaiya K, Feskens EJ (2015). Gestational diabetes mellitus in sub-Saharan Africa: systematic review and metaregression on prevalence and risk factors. Tropical Med Int Health.

[14] Larsen TB, Sorensen HT, Gislum M, Johnsen SP (2007). Maternal smoking, obesity, and risk of venous thromboembolism during pregnancy and the puerperium: a population-based nested case-control study. Thromb Res.

[15] Gaillard R, Steegers EA, Hofman A, Jaddoe VW (2011). Associations of maternal obesity with blood pressure and the risks of gestational hypertensive disorders. The generation R study. J Hypertens.

[16] Rahman MM, Abe SK, Kanda M, Narita S, Rahman MS, Bilano V, Ota E, Gilmour S, Shibuya K (2015). Maternal body mass index and risk of birth and maternal health outcomes in low- and middle-income countries: a systematic review and meta-analysis. Obes Rev.

[17] Young OM, Twedt R, Catov JM (2016). Pre-pregnancy maternal obesity and the risk of preterm preeclampsia in the American primigravida. Obesity (Silver Spring).

[18] Vinturache A, Moledina N, McDonald S, Slater D, Tough S (2014). Pre-pregnancy Body Mass Index (BMI) and delivery outcomes in a Canadian population. BMC Pregnancy Childbirth.

[19] Cnattingius S, Villamor E, Johansson S, Bonamy AE, Persson M, Wikström A, Granath F (2013). Maternal obesity and risk of preterm delivery. JAMA.

[20] Moore L, Singer M, Bradlee M, Rothman K, Milunsky A (2000). A prospective study of the risk of congenital defects associated with maternal obesity and diabetes mellitus. Epidemiolog.

[21] Martínez-Frías M, Frías J, Bermejo E, Rodríguez-Pinilla E, Prieto L, Frías J (2005). Pre-gestational maternal body mass index predicts an increased risk of congenital malformations in infants of mothers with gestational diabetes. Diabet Med.

[22] Ehrenberg HM, Mercer BM, Catalano PM (2004). The influence of obesity and diabetes on the prevalence of macrosomia. Am J Obstet Gynecol.

[23] Shoar Z, Zivot A, Nasiri S, Mandhani N, Kelly B. Maternal obesity, maternal gestational diabetes mellitus, and maternal and neonatal outcomes. J Obes Weight Loss Therapy. 2016;06(01):1-12.

[24] Wendland EM, Torloni MR, Falavigna M, Trujillo J, Dode MA, Campos MA, Duncan BB, Schmidt MI (2012). Gestational diabetes and pregnancy outcomes--a systematic review of the World Health Organization (WHO) and the International Association of Diabetes in Pregnancy Study Groups (IADPSG) diagnostic criteria. BMC Pregnancy Childbirth.

[25] Byrn M, Penckofer S (2015). The relationship between gestational diabetes and antenatal depression. J Obstet Gynecol Neonatal Nurs.

[26] Katon JG, Russo J, Gavin AR, Melville JL, Katon WJ (2011). Diabetes and depression in pregnancy: is there an association?. J Women's Health (Larchmt).

[27] Checkley W, Ghannem H, Irazola V, Kimaiyo S, Levitt NS, Miranda JJ, Niessen L, Prabhakaran D, Rabadan-Diehl C, Ramirez-Zea M (2014). Management of NCD in low- and middle-income countries. Glob Heart.

[28] Nabel EG, Stevens S, Smith R (2009). Combating chronic disease in developing countries. Lancet.

[29] Informática INdEse (2015). Peru Enfermedades No transmísibles y Transmisibles 2014.

[30] Metzger BE, Gabbe SG, Persson B, Buchanan TA, Catalano PA, Damm P, Dyer AR, Leiva A, International Association of D, Pregnancy Study Groups Consensus P (2010). International association of diabetes and pregnancy study groups recommendations on the diagnosis and classification of hyperglycemia in pregnancy. Diabetes Care.

[31] Wulsin L, Somoza E, Heck J (2002). The feasibility of using the Spanish PHQ-9 to screen for depression in primary care in Honduras. Prim Care Companion J Clin Psychiatry.

[32] Kroenke K, Spitzer R, Williams J (2001). The PHQ-9: validity of a brief depression severity measure. J Gen Intern Med.

[33] Zhong Q, Gelaye B, Fann JR, Sanchez SE, Williams MA (2014). Cross-cultural validity of the Spanish version of PHQ-9 among pregnant Peruvian women: a Rasch item response theory analysis. J Affect Disord.

[34] Rothman KJ, Greenland S, Lash TL: Modern Epidemiology. Philadelphia: Lippincott Williams & Wilkins; 2008.

[35] Sacks D, Hadden D, Maresh M, Deerochanawong C, Dyer A, Metzger B, Lowe L, Coustan D, Hod M, Oats J (2012). Frequency of gestational diabetes mellitus at collaborating centers based on IADPSG consensus panel-recommended criteria: the Hyperglycemia and Adverse Pregnancy Outcome (HAPO) study. Diabetes Care.

[36] Lavery JA, Friedman AM, Keyes KM, Wright JD, Ananth CV. Gestational diabetes in the United States: temporal changes in prevalence rates between 1979 and 2010. BJOG. 2017;24(5):804-13. 10.1111/1471-0528.14236 PMC5303559 27510598

[37] Koo BK, Lee JH, Kim J, Jang EJ, Lee CH (2016). Prevalence of gestational diabetes mellitus in Korea: a National Health Insurance Database Study. PLoS One.

[38] Kumari A (2001). Pregnancy outcome in women with morbid obesity. Int J Gynaecol Obstet.

[39] Bianco A, Smilen S, Davis Y, Lopez S, Lapinski R, Lockwood C (1998). Pregnancy outcome and weight gain recommendations for the morbidly obese woman. Obstet Gynecol.

[40] Chu SY, Callaghan WM, Kim SY, Schmid CH, Lau J, England LJ, Dietz PM (2007). Maternal obesity and risk of gestational diabetes mellitus. Diabetes Care.

[41] Kim SY, England L, Sappenfield W, Wilson HG, Bish CL, Salihu HM, Sharma AJ (2012). Racial/ethnic differences in the percentage of gestational diabetes mellitus cases attributable to overweight and obesity, Florida, 2004-2007. Prev Chronic Dis.

[42] Group HSCR (2010). Hyperglycaemia and Adverse Pregnancy Outcome (HAPO) study: associations with maternal body mass index. BJOG.

[43] Hinkle SN, Buck Louis GM, Rawal S, Zhu Y, Albert PS, Zhang C (2016). A longitudinal study of depression and gestational diabetes in pregnancy and the postpartum period. Diabetologia.

[44] Barakat S, Martinez D, Thomas M, Handley M (2014). What do we know about gestational diabetes mellitus and risk for postpartum depression among ethnically diverse low-income women in the USA?. Arch Womens Ment Health.

[45] Ross GP, Falhammar H, Chen R, Barraclough H, Kleivenes O, Gallen I (2016). Relationship between depression and diabetes in pregnancy: a systematic review. World J Diabetes.

[46] O'Dwyer V, Farah N, Hogan J, O'Connor N, Kennelly MM, Turner MJ (2012). Timing of screening for gestational diabetes mellitus in women with moderate and severe obesity. Acta Obstet Gynecol Scand.

[47] Lutsiv O, McKinney B, Foster G, Taylor VH, Pullenayegum E, McDonald SD (2015). Pregnancy complications associated with the co-prevalence of excess maternal weight and depression. Int J Obes.

