# Supplementary material for: Prevalence and risk factors of gestational diabetes mellitus: findings from a universal screening feasibility program in Lima, Peru
Source: BMC Pregnancy Childbirth. 2018 Jul 18;18:303. doi: 10.1186/s12884-018-1904-0 (PMC6052534; doi:10.1186/s12884-018-1904-0)
Supplement: Supplementary file 1 [file 12884_2018_1904_MOESM1_ESM.docx]

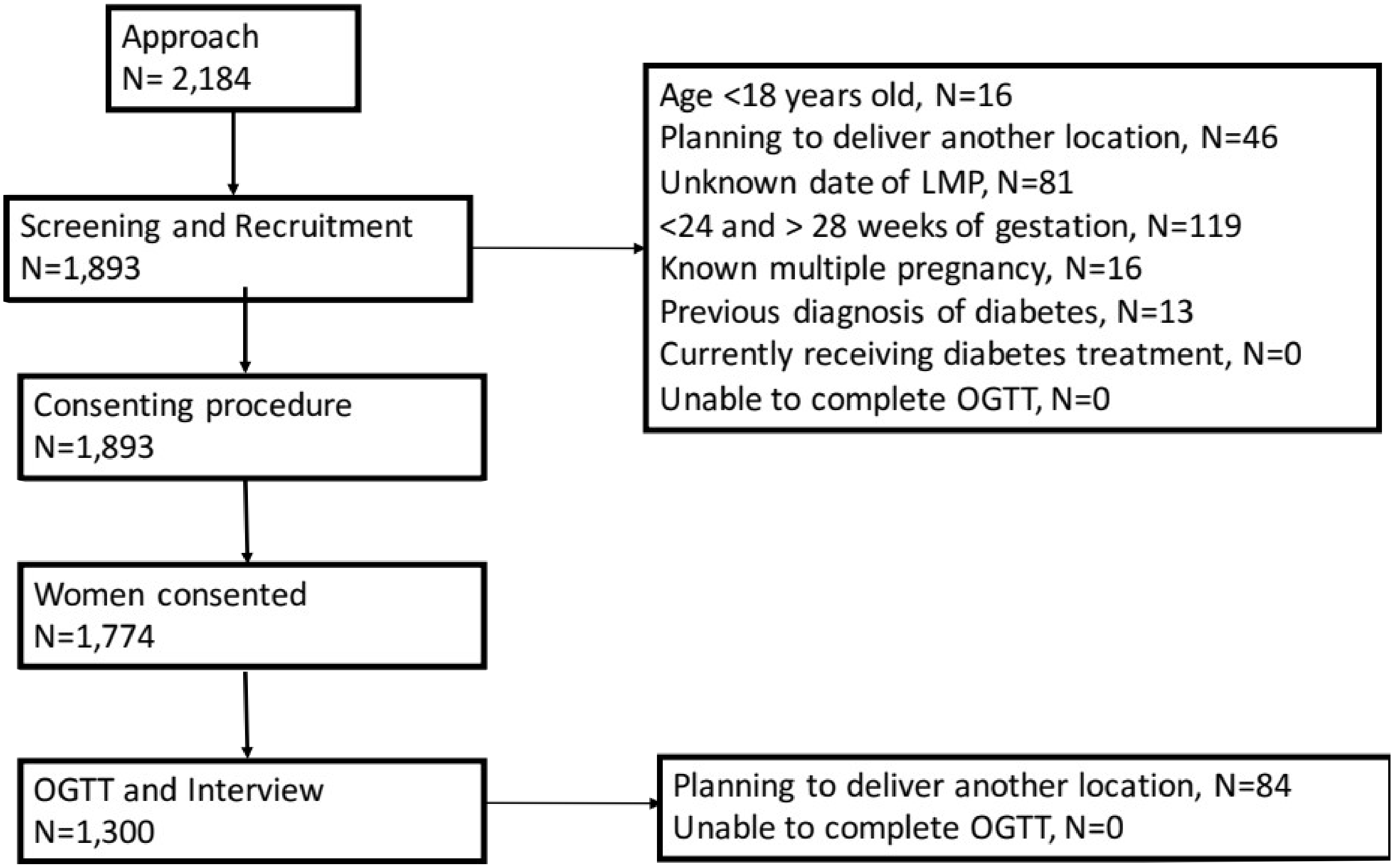
Approach
N= 2,184
Screening and Recruitment
N=1,893
Age <18 years old, N=16
Planning to deliver another location, N=46
Unknown date of LMP, N=81
<24 and > 28 weeks of gestation, N=119
Known multiple pregnancy, N=16
Previous diagnosis of diabetes, N=13
Currently receiving diabetes treatment, N=0
Unable to complete OGTT, N=0
Consenting procedure
N=1,893
Women consented
N=1,774
OGTT and Interview
N=1,300
Planning to deliver another location, N=84
Unable to complete OGTT, N=0
